# Robust Tomato Recognition for Robotic Harvesting Using Feature Images Fusion

**DOI:** 10.3390/s16020173

**Published:** 2016-01-29

**Authors:** Yuanshen Zhao, Liang Gong, Yixiang Huang, Chengliang Liu

**Affiliations:** State Key Laboratory of Mechanical System and Vibration, Shanghai Jiao Tong University, Shanghai 200240, China; zhaoyuanshen@126.com (Y.Z.); gongliang_mi@sjtu.edu.cn (L.G.); huang.yixiang@sjtu.edu.cn (Y.H.)

**Keywords:** tomato recognition, robotic harvesting, low cost, image fusion, multiple feature images

## Abstract

Automatic recognition of mature fruits in a complex agricultural environment is still a challenge for an autonomous harvesting robot due to various disturbances existing in the background of the image. The bottleneck to robust fruit recognition is reducing influence from two main disturbances: illumination and overlapping. In order to recognize the tomato in the tree canopy using a low-cost camera, a robust tomato recognition algorithm based on multiple feature images and image fusion was studied in this paper. Firstly, two novel feature images, the  a*-component image and the I-component image, were extracted from the L*a*b* color space and luminance, in-phase, quadrature-phase (YIQ) color space, respectively. Secondly, wavelet transformation was adopted to fuse the two feature images at the pixel level, which combined the feature information of the two source images. Thirdly, in order to segment the target tomato from the background, an adaptive threshold algorithm was used to get the optimal threshold. The final segmentation result was processed by morphology operation to reduce a small amount of noise. In the detection tests, 93% target tomatoes were recognized out of 200 overall samples. It indicates that the proposed tomato recognition method is available for robotic tomato harvesting in the uncontrolled environment with low cost.

## 1. Introduction

The tomato is one of the most important agricultural products around the world, and China has become one of the world’s largest tomato producers since 1995, according to Food and Agriculture Organization of the United Nations Statistics Database (FAOSTAT) [1]. Tomato harvesting requires a large amount of labor; however, a labor shortage emerged in the agricultural industry worldwide, especially in China. The development of a tomato harvesting robot is an effective way to address the labor shortage and high labor cost. To date, the commercial application of fruit harvesting robots is still unavailable because of the robotic harvesting inefficiencies and the lack of economic justification. Since the 1960s, Schertz *et al.* [2] proposed the concept of the autonomous harvesting robot; the major technical challenges to robotic harvesting can be summarized in three issues: fruit recognition [3,4], hand-eye coordination [5,6], and end-effecter design [7]. A number of achievements in the hand-eye coordination and end-effector design have been reported in this literature [8,9,10]. The more complex bottleneck to improving the efficiency of the harvesting robot is recognizing the target fruit in the uncontrolled environment [11,12,13,14].

In the study of the first fruit harvesting robot in the 1980s, a black and white camera was applied to detect apples in the tree canopy [15]. With the development of sensor technology, many kinds of visual sensors have been used in the fruit recognition systems of harvesting robots. Digital cameras such as CCD (Charge Coupled Device) and CMOS (Complementary Metal Oxide Semiconductor) are the most commonly used sensor in fruit recognition. On the other hand, some researchers have used novel visual sensors such as the structure light system, hyperspectral camera, and thermal camera for fruit recognition. Tankgaki *et al.* [16] adopted a structure light system which was equipped with red and infrared lasers to detect the fruit in the tree. Okamoto *et al.* [17] developed a green citrus recognition method using a hyperspectral camera of 369–1042 nm. The fruit detection tests revealed that 80%–89% of the fruit in the foreground of the validation set were identified correctly. Bulanon *et al.* [18] used a thermal infrared camera to improve the detection rate of citrus in the tree canopy for robotic harvesting. Additionally, the combination of multi-sensors may achieve a better performance of fruit detection and localization [19,20,21]. Even though these technologies can provide better recognition results, these recognition systems require expensive instruments. Thus, these technologies are not suitable for practical use. It is desirable to develop a fruit recognition method which can get a certain accuracy using the low-cost cameras. 

In this paper, a robust tomato recognition method using RGB images is developed. The proposed method is based on multiple feature images and image fusion, which is available for robotic tomato harvesting in uncontrolled senses. Two novel feature images were extracted from multiple color spaces which are transformed from the RGB images. Then, the two feature images are fused according to the image fusion strategy of wavelet transformation. The fusion images can reduce the influence of disturbances such as varying illumination and overlapping. Finally, an adaptive threshold algorithm is used to segment the target fruit from the complex background. In the detection tests, 200 samples (images) with two main disturbances, varying illumination and overlapping, are used to assess the performance of the proposed algorithm.

## 2. Materials and Methods

### 2.1. Image Acquisition

Tomato images were collected in the tomato planting greenhouses of the Sunqiao Modern Agricultural Park in Shanghai. The image acquisition systems mainly consisted of the following hardware: a computer (Lenovo, Beijing, China, nter(R) Core(TM) i3-370 CPU, Random Access Memory (RAM) 4.0GB) and a Complementary Metal Oxide Semiconductor (CMOS) camera (model ID MER-500-7UC, DAHENG IMAVISION, Beijing, China) with 388 × 260 pixel. All the images were taken under natural daylight conditions, which included two typical disturbances: varying illumination and overlapping. A total of 200 samples (tomato images) were acquired, and the number of samples with each type of disturbances was 100.

### 2.2. Feature Image Extraction

There are many studies aimed at recognizing the target fruit in the background using color feature extraction [22]. In their studies, the target fruit was removed from the background in a special color space such as RGB (red, green, blue) and HSV (hue, saturation, value) color space. Arman *et al.* proposed a ripe tomato recognition method by extracting the feature images from RGB, hue, saturation, intensity (HSI), and luminance, in-phase, quadrature-phase (YIQ) color spaces. Huang *et al.* also studied the automatic recognition of ripe Fuji apples in tree canopy using three distinguishable color models which were L*a*b*, HSI, and Liquid Crystal Display (LCD) color spaces. They conducted threshold segmentation in three different color spaces. According to the recent studies, two feature images which are a*-component image and I-component image are extracted from YIQ color space and L*a*b* color space, respectively.

#### 2.2.1. a*-**C**omponent Image

L*a*b* color space is a three-dimensional color model consisting of chrominance and brightness. It is a group of feature images (L*, a*, b*), which is available to all light colors and object color representation or calculation. In the tree feature images, a*-component describes the distribution of colors from red to green. Because a*-component images are independent of brightness, the a*-component image can be used to reflect the color characteristics of the ripe tomatoes. So the a*-component image is selected as one of the source images for fusion. The conversion relationship between L*a*b* color space and RGB color space is nonlinear, which needs to transform from RGB color space to XYZ color space as shown in Equation (1).

(1) $$\left[\begin{array}{c} X\\ Y\\ Z \end{array}\right]=\left[\begin{array}{ccc} 0.433953 & 0.376219 & 0.189828\\ 0.212671 & 0.715160 & 0.072169\\ 0.017758 & 0.109477 & 0.872765 \end{array}\right]\times \left[\begin{array}{c} r\\ g\\ b \end{array}\right]$$

where *X*, *Y* and *Z* are the features of the XYZ space, and r, g and b are normalized results of *R*, *G* and *B* which are the three primary colors of RGB space. The conversion formula is given as follows:

(2) $$\{\begin{array}{c} r=R/\left(R+G+B\right)\\ g=G/\left(R+G+B\right)\\ b=B/\left(R+G+B\right) \end{array}$$

The L*a*b* color space can be transformed from XYZ color space through Equation (3), in which the intermediate function f(t), as shown in Equation (4), has two kinds of expressions according to the value of *t*. Using Equations (1)–(4), the a*-component can be extracted.

(3) $$\{\begin{array}{c} L*=116\times f(Y)-16\\ a*=500\times (f(X)-f(Y))\\ b*=200\times (f(Y)-f(Z)) \end{array}$$

(4) $$f(t)=\{\begin{array}{ll} t^{1/3} & t>0.008856\\ f(t)=7.787\times t+16/116 & t\leq 0.008856 \end{array}$$

where L*, a* and b* are the features of L*a*b* color space, and *X*, *Y* and *Z* have the same meanings as in Equation (1). 

#### 2.2.2. I-Component Image

YIQ color space is proposed by the U.S. National Television Standards Committee (NTSC) for improving the image recognition performance. YIQ color space which consists of three component images reflects the brightness of the image and the feature of color range, respectively. I-component image is also selected as the other source image for fusion. The conversion relationship between YIQ color space and RGB is linear, and the I-component image can be obtained according to the transformation formula which is given as follows:

(5) $$\{\begin{array}{c} Y=0.2990\times R+0.5870\times G+0.1140\times B\\ I=0.5957\times R-0.2745\times G-0.3213\times B\\ Q=0.2115\times R-0.5226\times G+0.3111\times B \end{array}$$

where *R*, *G*, and *B* have the same meanings as in Equation (1); *Y*, *I*, *Q* are the feature components of YIQ color space.

### 2.3. Image Fusion Strategy

**Figure 1:**
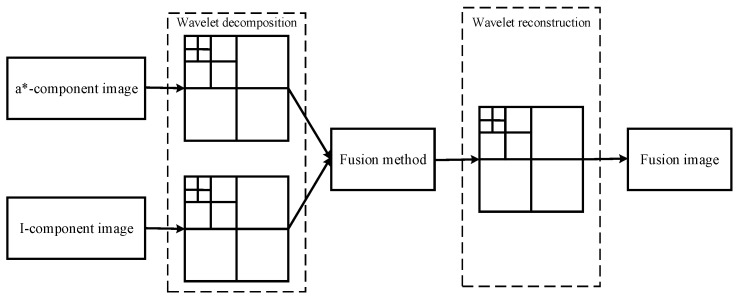
Strategy of image fusion based on wavelet transform.

The purpose of image fusion is the combination of the information features from the source images. The common methods used for image fusion are multi-scale image analysis, and wavelet transformation is one of the most classic methods for image fusion [12]. In this paper, wavelet transformation is employed to fuse the a*-component image and I-component image at pixel level. The strategy of image fusion is shown in Figure 1. 

Compared with these fruit recognition methods based on multi-sensor fusion, the superiority of the algorithm proposed in this paper is lower time-consumption which is not necessary for image registration. Because of the source images being transformed from the same picture, the source images can be used for image fusion directly. As shown in Figure 1, the a*-component image and I-component image are input as the source images which are needed to convert into the form of a matrix. To acquire the wavelet decomposition coefficients *C*1 and *C*2, the a*-component image and I-component image are decomposed based on wavelet transformation, respectively. The image fusion principle is aimed at getting the fusion coefficient. Firstly, compare the values of two image matrices. Secondly, define the larger one as the matrix *L*_max_. Thirdly, compute the matrix *L*_max_ to get the minimum element *x*_min_ and the maximum *x*_max_. The intermediate variable *d* is defined as the difference between *x*_max_ and *x*_min_,

(6) $$d=x_{\max}-x_{\min}$$

where *x*_max_ and *x*_min_ are the maximum and minimum elements of the matrix *L*_max_. Additionally, the fusion coefficient is obtained through Equation (7).

(7) C=(1−d)×C1+d×C2

where *C* is the fusion coefficient of the wavelet transformation; *C*1 and *C*2 are the wavelet decomposition coefficients of two source images, respectively. Finally, the fusion image can be obtained through the wavelet reconstruction.

### 2.4. Adaptive Threshold Segmentation 

In order to extract the target tomato from the fusion images, an adaptive threshold segmentation algorithm is given in this paper. The key of this algorithm is to determine the appropriate threshold automatically. The detailed steps are as follows:

(1) Assume the resolution of the fusion image is M × N; if a pixel on the fusion image is recorded as (i, j), then the grayscale value of the pixel (i, j) is recorded as *T*_(i, j)_;
(2) Calculate the maximum and the minimum grayscale values of the fusion image: *T*_max_ and *T*_min_. The initial threshold can be worked out according to the following formula:
  (8) $$T^{k}=\frac{T_{\max}+T_{\min}}{2}$$
  where *k* is the number of iterations and *T^k^* is the grayscale result after *k* iterations.
(3) Based on the grayscale *T^k^*, the fusion image is divided into two groups, A and B, and the average grayscale of A and B areas can be calculated by Equation (9).
  (9) $$\{\begin{array}{c} T_{A}=\sum_{i=0,j=0}^{M,N}\frac{T_{\left(i,j\right)}}{W}(T_{\left(i,j\right)}>T^{k})\\ T_{B}=\sum_{i=0,j=0}^{M,N}\frac{T_{\left(i,j\right)}}{1-W}(T_{\left(i,j\right)}<T^{k}) \end{array}$$
  where *T_A_* and *T_B_* are the average grayscale of A and B areas and the W is the number of pixels that have grayscales that are larger than *T^k^*.
(4) After k times iterations, the new threshold is calculated as follows:
  (10) $$T^{k+1}=\frac{T_{A}+T_{B}}{2}$$
(5) Repeat steps (3) and (4), until *T^k^* = *T^k^* ^+ 1^; and take the result *T^k^*^+ 1^ as the final threshold *T*_m_.
(6) Obtain the other threshold *T*_n_ by the Otsu algorithm.
(7) Determine the final segmentation threshold on the basis of comparing the results of *T*_m_ and *T*_n_. When *T*_m_ is equal to and larger than *T*_n_, define *T*_n_ as the final threshold *T*_f_; when *T*_m_ is less than *T*_n_, define *T*_m_ as the final threshold *T*_f_.
(8) The fusion image segmentation is calculated according to Equation (11), which is also defined as binary image processing.
  (11) $$T_{\left(i,j\right)}=\{\begin{array}{c} 1\begin{array}{ccc} & & \left(T_{\left(i,j\right)}\leq T_{f}\right) \end{array}\\ 0\begin{array}{ccc} & & \left(T_{\left(i,j\right)}>T_{f}\right) \end{array} \end{array}$$

### 2.5. Morphology Operation

There may be a small amount of noise that still exists in results of image segmentation. Through the morphology operation, the noises in the segmented image can be removed. According to the experiment results, the pixel area of threshold is selected as 200. So, if the pixel area of the extracted object is less than 200 pixels, it is considered noise and it needs to be removed. 

**Figure 2:**
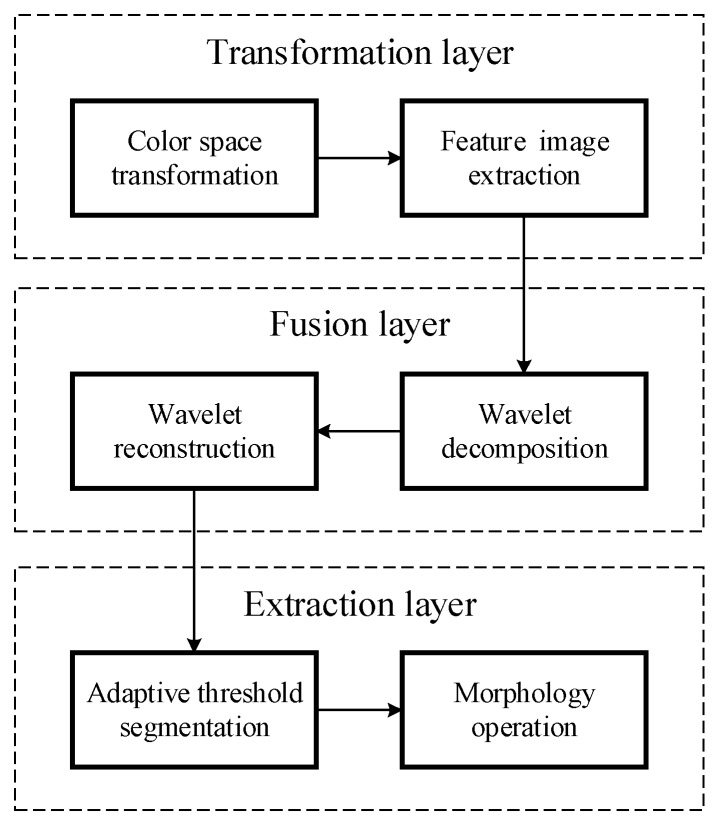
Overview of the automated tomato recognition method.

Based on the analysis above, a complete and detailed program of the robust tomato recognition algorithm is shown in Figure 2. It is divided into three layers: the transformation layer, fusion layer, and extraction layer. The transformation layer includes color space transformations and feature image extraction. The fusion layer is built based on wavelet transformation. The extraction layer consists of adaptive threshold segmentation and morphology operation.

## 3. Results and Discussion

### 3.1. Tomato Recognition 

**Figure 3:**
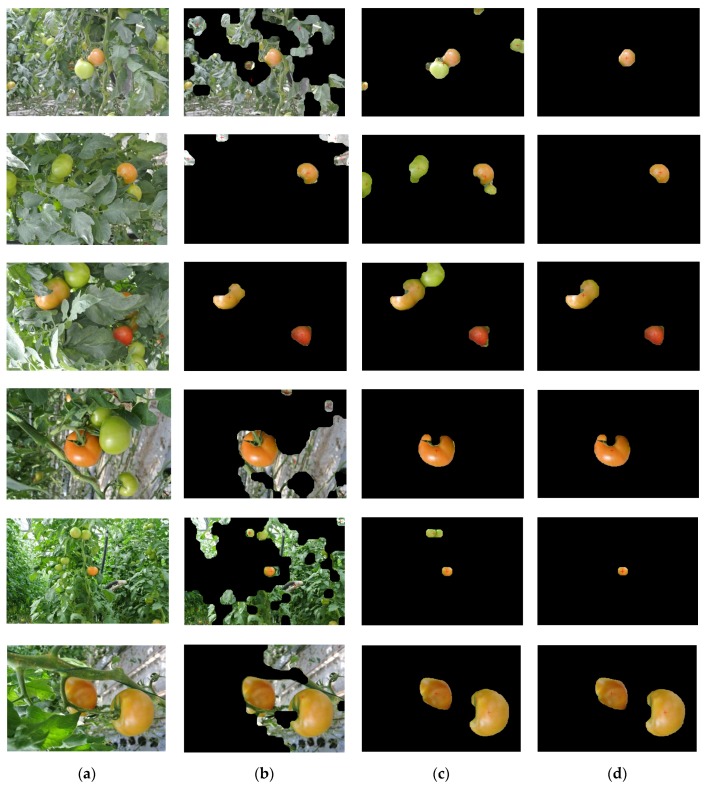
Examples of the tomato recognition. Images in column (**a**) show the original images with tomatoes in plant canopies. Images in column (**b**), (**c**) and (**d**) show the results of three recognition methods which are using a*-component images, I-component images and fusion images, respectively.

Considering the complex natural agriculture environment, the varying illumination and overlapping are the main factors influencing the results of tomato recognition. So, the two disturbances are taken into account in the image acquisition. A total of 200 tomato images are divided into two groups for contrast tests. Each group of the tests includes 100 tomato images with one of two typical disturbances as samples. The contrast tests are conducted between the feature images (a*-component image and I-component image) [23] and the fusion image. Both the fusion image and source images are processed by the improved threshold segmentation method which was proposed in this paper. To compare and assess the performance of recognition methods, examples of the contrast test results using the a*-component image, I-component image and fusion image, respectively, are shown in Figure 3. 

The results of the three recognition approaches when applied to 200 testing images are presented in Table 1. It indicates that the recognition rate of the fusion image is about 93% in general, while the recognition accuracies of the a*-component image and I-component image were 56% and 63%, respectively. The proposed approach shows that the recognition accuracy was increased compared to the conventional approach of detection using the a*-component image or I-component image alone.

**Table 1:** Performance analysis of different recognition algorithms.

Types of Disturbances	Total	Recognized		
		a*-component Image	I-component Image	Fusion Image
variable illumination	100	44	67	97
clustering of immature fruit	100	68	59	89

### 3.2. Histogram Analysis

**Figure 4:**
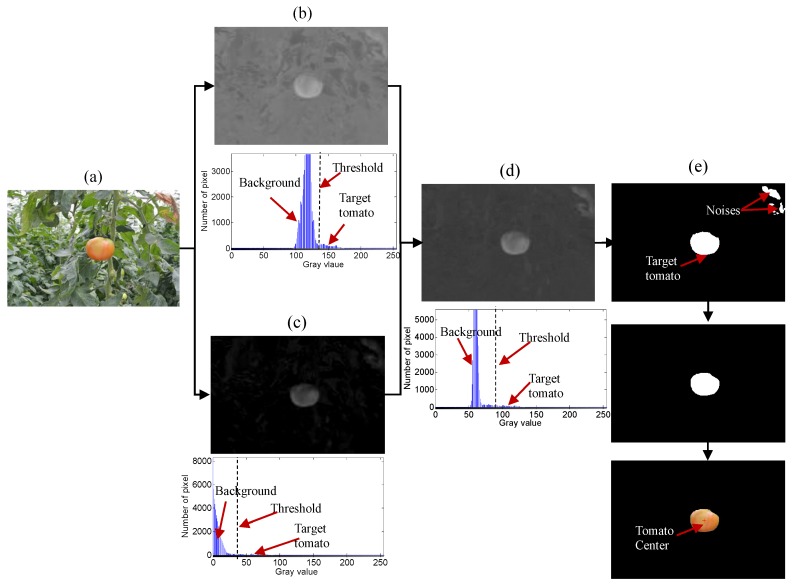
Example of histogram analysis by image fusion. (**a**) Original RGB image; (**b**) a*-component image and its histogram; (**c**) I-component image and its histogram; (**d**) Fusion image and its histogram; (**e**) Result of tomato recognition.

The histogram analysis results are shown in Figure 4. The original RGB image includes a mature tomato and complex agricultural background. The complex agricultural background consists of the leaves and stems of the plants, greenhouse system, and the other disturbances. Through color space transformation, the a*-component image and I-component image can be extracted and their gray distribution can also be shown in the histogram. The gray value of the I-component image ranges from 110 to 170, and its threshold is about 142, while the gray distribution of the a*-component image is from 0 to 80, and its threshold is about 38. As shown in Figure 4, the gray distribution of the fusion image changes obviously. According to the histogram analysis, the gray distribution of the fusion image is from 50 to 130. So, the threshold for segmentation is about 90 through the adaptive threshold calculated. The gray distributions of the a*-component image, I-component and fusion image are presented in Table 2.

**Table 2:** Values of the different thresholds processed by I-component image, a*-component image and fusion image.

Image	Gray Distribution	Threshold
a*-component image	100–170	140–145
I -component image	0–80	25–50
Fusion image	50–130	80–100

In special scenes, the fusion image does not have enough information to perform an accurate identification. As shown in Figure 4, there are some noises that still exist in the segmentation result. That is because the dried leaves have the same gray value as the tomato in the fusion image. Therefore, morphology operation is necessary for removing the noise in the binary image. If the pixel area of the segmentation object is less than 200, the object is regarded as noise. After the image process of segmentation and de-noising, the target tomato is detected.

### 3.3. Comparative Analysis

The wavelet transformation-based image fusion is aimed at acquiring a single image which can provide more precision and more comprehensive information about the same scene. In this study, the pixel level fusion was needed to three-level wavelet decompose at first. Additionally, the fusion image was finally obtained with the processing of the wavelet reconstruction. For enhancing the advantages of the proposed algorithms, a group of comparative tests between the proposed algorithm using wavelet transformation and the simple fusion strategy proposed in the literature [24] was conducted. The simple fusion strategy used a simple combination of the a*-component image and I-component image directly.

**Figure 5:**
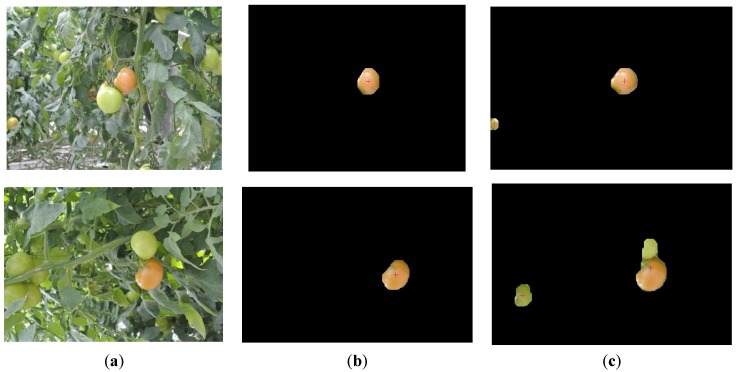
The results of comparative tests between proposed approach and the simple fusion strategy: (**a**) Original images; (**b**) Results of proposed algorithm; (**c**) Results of simple combination.

The results of the comparative tests were shown in Figure 5. The test results indicated that the performance of the proposed algorithm based on wavelet transformation was better than the simple fusion strategy. As shown in Figure 5c, the detection results of the simple combination approach could include some false positives. In other words, some immature tomatoes were misidentified as the target tomatoes which existed in the detection results. However, the proposed approach using wavelet transformation not only detected the target tomatoes but also made no misidentification. 

### 3.4. Robust Analysis

The performance analysis is conducted by the three recognition methods which are employed for the proposed image processing of adaptive threshold segmentation and morphology operation. However, the difference between the three algorithms is whether or not they include fusion layers. The comparative experiments can show the advantage of the image fusion which is the core of the algorithm proposed in this paper.

#### 3.4.1. Illumination Varying Influence

**Figure 6:**
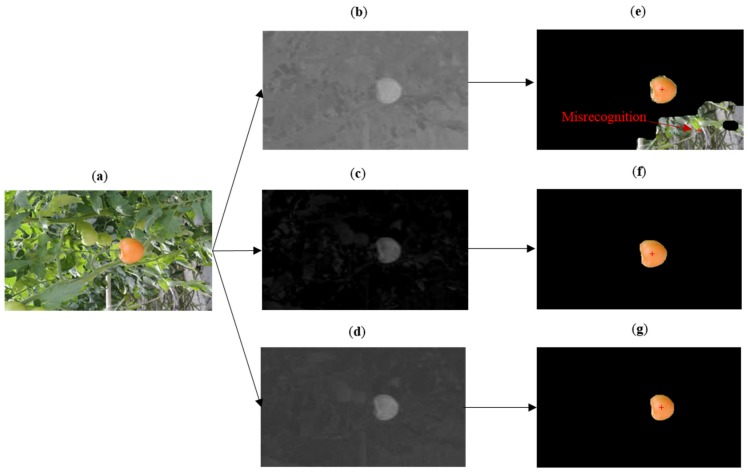
Contrast experiment on the disturbance of illumination. (**a**) Original image (illumination varying is obvious in the image); (**b**) a*-component image; (**c**) I-component image; (**d**) Fusion image of (**b**) and (**c**); (**e**) Recognition result of a*-component image; (**f**) Recognition result of I-component image; (**g**) Recognition result of fusion image.

Figure 6 reflects that the fusion image and I-component image have the better performances in the condition of varying illumination. In the RGB image, except for the mature tomato, the background also includes many disturbances such as leaves, stems and the ground, while the main disturbance for recognizing the tomato is the strong sunlight. The a*-component image and I-component image are transformed from the RGB image. As shown in Figure 6, the a*-component image has a low discrimination of pixel value between the target tomato and background. However, the I-component image and fusion image have a remarkable discrimination between the target tomato and background. So, through adaptive threshold segmentation, the segmentation results of the fusion image and I-component image can detect the mature tomato precisely. Because of the influence of the illumination, the segmentation threshold of the a*-component image is not the optimum value. The partial region pixels which have gray values that are similar to the gray values of the target tomato are still kept in the recognition result. So, it indicates that the recognition algorithm proposed in this paper has the advantage of reducing the negative effect of illumination in tomato recognition.

#### 3.4.2. Overlapped Influence

**Figure 7:**
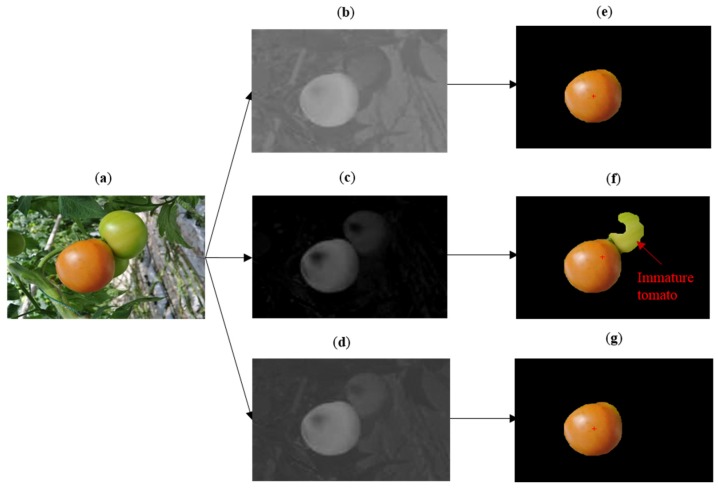
Contrast experiment about the disturbance of overlapping tomatoes. (**a**) Original image (tomatoes are overlapped in the image ); (**b**) a*-component image; (**c**) I-component image; (**d**) Fusion image of (**b**) and (**c**); (**e**) Recognition result of a*-component image; (**f**) Recognition result of I-component image; (**g**) Recognition result of fusion image.

Figure 7 indicates that the a*-component image and fusion image have precise recognition results when the target tomato is overlapped by the immature tomatoes. As shown in the RGB image, there are many disturbances such as leaves, stems, and greenhouse equipment that exist in the picture. However, the main interference factor for tomato recognition is the overlapping by immature tomatoes. The a*-component image, I-component image and fusion image are obtained respectively, and the segmentation results of the three images have different performances. The fusion image and a*-component image achieved better recognition performance, while the immature tomato was misidentified in the process of recognizing the I-component image. It successfully demonstrates that the recognition algorithm based on the fusion of the a*-component image and I-component image can recognize the mature tomato which is overlapped by the immature tomatoes.

Moreover, with the different types of disturbances, the recognition rates of the three methods are also different from each other. The I-component images and fusion images have higher recognition rates than the I-component images when varying illumination is the main disturbance. When the main disturbance is overlapping, the fusion images and a*-component images show better performances than the single I-component images. The reason for the experiment results is that the fusion images have combined the feature information of the a*-component images and I-component images. Even though several samples were not recognized, the success rate of the recognition method using the low-cost vision sensor is very encouraging. Therefore, the proposed recognition algorithm is available for robotic tomato harvesting.

## 4. Conclusions

In this paper, a robust tomato recognition method using a low-cost camera was developed. Compared with the single feature image recognition methods, the proposed recognition approach can enhance the feature information of the target tomato through the fusion of multiple color space feature images. Without image registration, the proposed method can reduce the time-consumption, which is a key technical index for a fruit harvesting robot. On the other hand, the bottleneck of fruit recognition is poor adaptability. Especially varying illumination and overlapping by immature tomatoes are the main influences on the adaptability of the recognition algorithm. Different from the typical recognition algorithms, the tomato recognition algorithm proposed in this paper can meet the challenge of these two main disturbances.

Two hundred groups of comparison experiments were conducted to show the advantages of the proposed tomato recognition method. The results demonstrate that the I-component image is superior in reducing the negative effects caused by varying illumination. Meanwhile, the a*-component image owns feature information which can reflect the gray distribution between the target tomato and background. So, the image fusion from the a*-component image and I-component image combined the feature information of the two source images, and this enhances the performance of tomato recognition. The success recognition rate of the proposed method is 93% in general, which is available for robotic tomato harvesting. The next work would be focused on integrating the proposed methods into the autonomous tomato harvesting robot. 
